# A Case of a One-Month-Old Infant Who Underwent Oral Intubation With Tongue Traction After Decompressing an Oral Cyst

**DOI:** 10.7759/cureus.79132

**Published:** 2025-02-17

**Authors:** Takehito Sato, Yui Somura, Masashi Takakura, Takahiro Tamura, Shogo Suzuki

**Affiliations:** 1 Department of Anesthesiology, Nagoya University Hospital, Nagoya, JPN

**Keywords:** difficult airway management, infant, intubation, oral ranula, video laryngoscopy (vl)

## Abstract

Lymphangioma is a rare congenital anomaly of the lymphatic system that can lead to an airway emergency when it occurs in the oral cavity or neck. Here, we report a case of a one-month-old infant who presented with an airway emergency due to a giant lymphangioma. The infant was rushed to the ICU with respiratory and feeding difficulties, and examination revealed a large mass on the floor of the mouth. Airway management was challenging due to the cyst. After puncturing the cyst, a suture was placed at the tip of the tongue to allow forward traction. The oral surgeon then pulled the tongue forward to secure space for laryngoscope insertion. Awake intubation was performed with 0.03 mg of atropine, and a McGrath Mac Disposable Laryngoscope Blade #1 (Aircraft Medical, Edinburgh, Scotland) was inserted under spontaneous respiration.

Tongue traction and video laryngoscopy were used for intubation, and this method may be considered a valuable option for airway management.

## Introduction

Lymphangiomas are rare congenital malformations of the lymphatic system, affecting 1 in 6,000-16,000 newborns [1]. Approximately 75% of lymphangiomas occur in the head and neck regions. When they develop in the oral cavity or neck, they can lead to airway emergencies [2,3].

Respiratory distress due to lymphangiomas can progress rapidly, necessitating immediate interventions to secure the airway. If respiratory distress worsens, emergency intubation may be required. Additionally, the unique anatomy and physiology of infants pose significant challenges for clinicians [4].

Herein, we report a case of a one-month-old infant who presented with an airway emergency due to a giant lymphangioma, requiring innovative approaches to secure the airway.

## Case presentation

Written informed consent was obtained from the guardians for this case report.

The patient was a one-month-old infant, weighing 4.1 kg and measuring 53 cm in height. A cyst was initially detected on the floor of the mouth at birth, leading to a referral for oral surgery due to a suspected ranula. The cyst was aspirated in the outpatient department, but it recurred and continued to grow.

**Figure 1:**
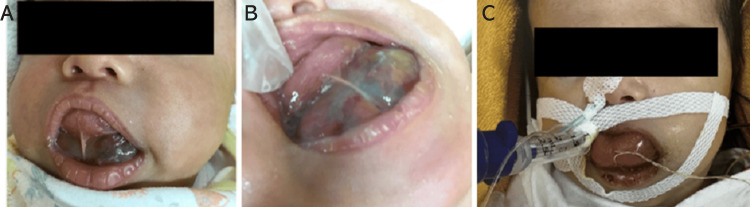
Facial appearance upon admission to the intensive care unit (A) A space-occupying lesion was present in the oral cavity, and the tongue was swollen with forward deviation, making mouth opening challenging. (B) Because of the space-occupying lesion, inserting a finger into the oral cavity was impossible. (C) After intubation, tongue edema was significant. The suture from which the tongue was retracted during intubation was placed on the tip of the tongue.

By the 40th day after birth, the cyst had enlarged, making breastfeeding difficult and causing desaturations, with percutaneous oxygen saturation dropping below 90%. As a result, the infant was admitted to the ICU. Upon examination, a cystic lesion on the floor of the mouth had enlarged, occupying the front of the oral cavity, restricting mouth opening, and causing the tongue to protrude (Figure 1A and 1B).

The airway was secured with a shoulder pillow, but oxygen saturation intermittently dropped below 90% due to breathing difficulties.

Intubation was deemed necessary, but the large cyst obstructed laryngoscope insertion. Therefore, we decided to puncture the cyst to reduce its size before intubation. After the cyst was punctured, a suture was placed at the tip of the tongue, allowing it to be pulled forward, creating space for the laryngoscope. Awake intubation was performed with 0.03 mg of atropine, and the McGrath Mac Disposable Laryngoscope Blade #1 was introduced under spontaneous breathing (Figure 1C).

**Figure 2:**
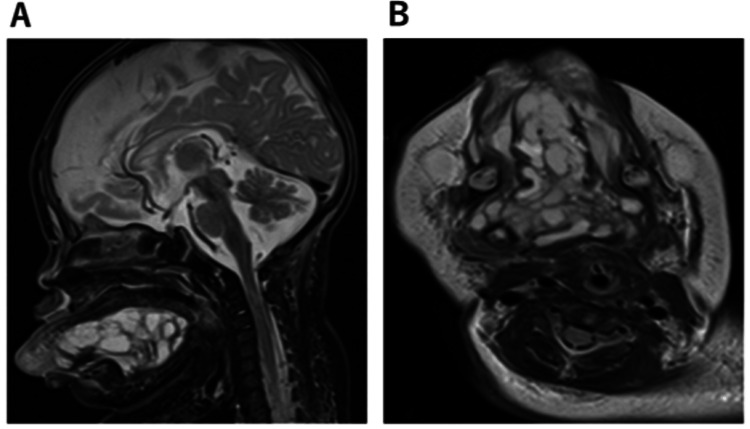
MRI (A) Sagittal plane on magnetic resonance imaging (MRI). (B) Horizontal plane on MRI. The figure shows a multilocular cystic mass, measuring approximately 6 × 5 × 3 cm in diameter, on the floor of the oral cavity and under the tongue.

**Figure 3:**
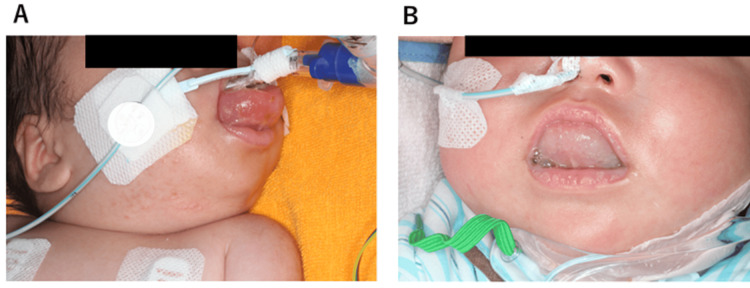
Facial appearances after sclerotherapy (A) After sclerotherapy with OK-432 (picibanil). Severe tongue edema was observed, and extubation was difficult. (B) Approximately two months after sclerotherapy, the lymphangioma on the floor of the mouth had decreased in size.

Magnetic resonance imaging revealed a multilocular cystic mass, approximately 6 × 5 × 3 cm in diameter, on the floor of the oral cavity and under the tongue (Figure 2). Histological analysis confirmed the diagnosis of lymphangioma. Therefore, sclerotherapy with OK-432 (picibanil) was administered on the fifth day of ICU admission. After sclerotherapy, intraoral and cervical edema worsened, and a tracheotomy was performed on the 28th day of ICU admission (Figure 3A).

The patient was discharged from the ICU on the 40th day. Two months later, the lymphangioma had reduced in size and showed no signs of recurrence. The patient was followed up as an outpatient (Figure 3B).

## Discussion

Airway management and intubation in infants can be challenging [3]. In adults, video laryngoscopes are commonly used for airway management [4]. In this case, the lesion occupying the oral cavity led to an airway emergency, necessitating airway security under spontaneous breathing. However, intubation was difficult due to the inability to insert the laryngoscope and the already unstable oxygenation. Given the risk of further airway obstruction, we chose to intubate the patient while preserving spontaneous breathing, avoiding sedation.

Few reports exist of difficult intubation due to intraoral lymphangioma in infants [5], and to date, the combination of video laryngoscopy with tongue traction in the surgical field for airway management has not been previously reported.

Awake or semi-awake intubation is commonly used in children with esophageal atresia [6]. These children lose spontaneous breathing, and positive pressure ventilation can lead to air entering the stomach through the tracheoesophageal fistula, causing poor ventilation and gastric rupture [7]. Therefore, awake intubation is required to preserve spontaneous breathing. Before intubation, sufficient oxygenation is provided, and atropine sulfate (0.01 mg/kg) is administered to prevent bradycardia.

In adults, awake nasal intubation, with or without light sedation, can be performed using a bronchoscope, especially when mouth opening is restricted [8]. This method ensures safe airway management while maintaining spontaneous breathing, as deep sedation is not required. Therefore, this method is generally effective.

However, awake nasal intubation in children is often more challenging due to their movement, narrow nasal passages, limited scope options, and fewer cases compared to adults [4,9].

Therefore, we decided to reduce the size of the lymphangioma by paracentesis to create space for intubation with video laryngoscopy. By retracting the tongue in the surgical field, we were able to insert the video laryngoscope blade, confirm the glottis on the screen, and, since the child was breathing spontaneously, insert the endotracheal tube during inhalation.

Thus, combining video laryngoscopy with tongue traction in the surgical field may be a valuable method for airway management in infants with intraoral masses.

## Conclusions

In conclusion, we report a case of a one-month-old infant presenting with an airway emergency caused by a giant lymphangioma. In infants with oral floor lymphangioma, securing the airway can be challenging and requires extreme care. A combination of tongue traction and video laryngoscopy was used for intubation.

This approach appears to be effective for securing the airway. It may be considered a valuable option for managing infants with giant intraoral masses.
